# Artificial Intelligence‐Guided Gut‐Microenvironment‐Triggered Imaging Sensor Reveals Potential Indicators of Parkinson's Disease

**DOI:** 10.1002/advs.202307819

**Published:** 2024-04-03

**Authors:** Yiwei Li, Hong‐Xia Ren, Chong‐Yung Chi, Yang‐Bao Miao

**Affiliations:** ^1^ Department of Haematology, Sichuan Academy of Medical Sciences & Sichuan Provincial People's Hospital School of Medicine of University of Electronic Science and Technology of China No. 32, West Section 2, First Ring Road, Qingyang District Chengdu 610000 China; ^2^ Institute of Communications Engineering & Department of Electrical Engineering National Tsing Hua University Hsinchu 30013 Taiwan; ^3^ Sichuan Technology & Business College Chengdu 611800 China

**Keywords:** artificial intelligence, deep learning, gut microbiota, oral delivery, sensor

## Abstract

The gut‐brain axis has recently emerged as a crucial link in the development and progression of Parkinson's disease (PD). Dysregulation of the gut microbiota has been implicated in the pathogenesis of this disease, sparking growing interest in the quest for non‐invasive biomarkers derived from the gut for early PD diagnosis. Herein, an artificial intelligence‐guided gut‐microenvironment‐triggered imaging sensor (Eu‐MOF@Au‐Aptmer) to achieve non‐invasive, accurate screening for various stages of PD is presented. The sensor works by analyzing α‐Syn in the gut using deep learning algorithms. By monitoring changes in α‐Syn, the sensor can predict the onset of PD with high accuracy. This work has the potential to revolutionize the diagnosis and treatment of PD by allowing for early intervention and personalized treatment plans. Moreover, it exemplifies the promising prospects of integrating artificial intelligence (AI) and advanced sensors in the monitoring and prediction of a broad spectrum of diseases and health conditions.

## Graphical abstract

An AI‐guided gut‐microenvironment‐triggered imaging sensor has been developed for non‐invasive PD diagnosis. By analyzing α‐Syn in the gut, this sensor accurately predicts the onset of PD, paving the way for early intervention diagnostic approaches. Beyond PD, this technology holds promise for monitoring and predicting a spectrum of diseases, highlighting the transformative potential of AI and advanced sensor technologies in healthcare.

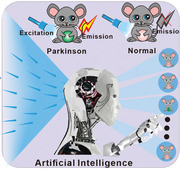

## 1 Introduction

Parkinson's disease (PD) is a long‐term degenerative disease of the central nervous system that primarily affects the motor system, and consequently causes most disability and death than other neurological disorders.^[^ ^1^, ^2^ ^]^ As a result, an opportunity window for treating PD may come up through the study of early diagnosis.^[^ ^3^, ^4^, ^5^ ^]^ However, the underlying etiology of PD remains elusive and still challenging research. Fortunately, recent pathological evidence, including clinical data, demonstrates a strong association between α‐Syn and PD, establishing it as a pivotal biomarker for both diagnosis and prognosis of the disease.^[^ ^6^, ^7^ ^]^ Furthermore, recent works discovered the fact that α‐Syn can be found in the gut before dwelling in the brain, thus lending credence to the theory that the pathophysiology of PD begins in the gut and then travels through the vagus nerve up to the brain.^[^ ^6^ ^]^ This discovery lays the ground for deploying a non‐invasive biological sensor to identify the various stages of PD.

In the context of non‐invasive tracking, oral delivery is often considered a promising approach due to its nature of user‐friendly, eliminating the need for needles, and promoting self‐administration.^[^ ^8^, ^9^ ^]^ However, the development of a non‐invasive gut‐microenvironment‐triggered biological sensor has been hampered by difficulties ranging from the instability of the imaging sensor in the stomach.^[^ ^10^ ^]^ Most biological sensors are rendered ineffective when exposed to gastric acid with a pH range of 1.0–3.0 and are subsequently broken down by proteases and deoxyribonucleases (DNase) found in the gastrointestinal tract (GI tract).^[^ ^11^ ^]^

To address the above challenges, it is proposed to immobilize biomacromolecules (aptamers), which are functional oligonucleotides capable of binding to specific target molecules, within an acid‐resistant, luminescent europium‐based metal‐organic framework (Eu‐MOF) with a precisely controlled pore size. Luminescent MOFs, which are crystalline porous cavities materials, consist of metal ions and organic ligands.^[^ ^12^, ^13^ ^]^ The luminescent Eu‐MOF can emit a bright fluorescence by the antenna effect.^[^ ^14^ ^]^ In the meantime, the aperture of luminescent MOFs can be manipulated to suit various applications, notably those involving the immobilization of bio‐macromolecules.^[^ ^15^ ^]^ Physical immobilization of the aptamer inside this pore cavity by luminescent Eu‐MOF produces armor that successfully protects the encapsulated aptamer under the hostile GI environment.^[^ ^16^ ^]^ Their pore size and surface charge are tailored to interact positively with aptamer and increase loading.^[^ ^17^ ^]^

**Figure 1:**
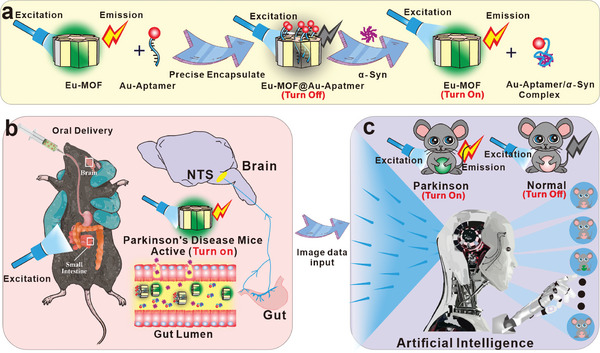
Composition/structure of artificial intelligence‐guided imaging sensor, and its operating mechanism. a) Composition and structure of the proposed gut‐microenvironment‐triggered imaging sensor (Eu‐MOF@Au‐Aptmer). b) After being administered orally, Eu‐MOF@Au‐Aptamer particles, which are able to identify α‐Syn, are then released into the gut in the form of Au‐Aptamer/α‐Syn complex. c) Proposed CNN model for predicting various stages of the PD mice from the acquired sensing data.

Therefore, a non‐invasive gut microenvironment‐triggered imaging sensor for the various stages surveillance of PD was developed and evaluated. This sensor employs a luminescent Eu‐MOF with a precise pore size loaded with an aptamer‐Au nanoparticle complex. Following the formation of the sensor, the fluorescence of the emission of Eu‐MOF can be quenched with Au‐Aptamer by fluorescence resonance energy transfer (FRET).^[^ ^18^, ^19^ ^]^ **Figure** **1**a illustrates the structure/composition of the proposed gut‐microenvironment‐triggered sensor and the methods by which it can be utilized for non‐invasive monitoring of PD via oral administration. We demonstrate that the Eu‐MOF matches the essential requirements of aptamer oral delivery materials, which include acid stability, appropriate pore size, aptamer protection under the gastrointestinal tract, and non‐invasive detection of PD. To guarantee the efficiency of aptamer loading into the pore size, the luminescent Eu‐MOF pore size (≈35 Å in diameter) can be big enough to accommodate aptamer that is between 22 and 26 Å in size,^[^ ^20^ ^]^ whereas it is sufficiently limited in size to inhibit the entry of deoxyribonuclease (DNase) (51.55, 61.07, and 104.47 Å).^[^ ^21^ ^]^ One important fact justified in this research is that hydrophobic contacts and/or electrostatic interactions between the aptamer and Eu‐MOF are effective for encapsulating Au‐Aptamer nanoparticles in luminescent Eu‐MOF via aperture.^[^ ^17^ ^]^ The protective environment within the pore of the sensor, when exposed to the harsh conditions of the stomach acid, minimizes excessive aptamer unfolding and thereby reduces degradation during oral administration (Figure 1b).^[^ ^22^ ^]^ Once α‐Syn are present in the GI tract, the Au‐Aptamer/α‐Syn complex is released into the GI tract via the capillary effect inherent in the proposed non‐invasive gut‐microenvironment‐triggered monitoring system.^[^ ^23^ ^]^ Upon removal of the Au‐aptamer nanoparticles from the system, a pronounced “turn‐on” fluorescence signal was observed in the PD mice, demonstrating the efficacy of the developed sensor.

To achieve accurate prediction of PD, this paper introduces a novel approach that leverages artificial intelligence (AI) and gut microenvironment‐triggered imaging sensors. AI plays a pivotal role in accurately predicting various stages of PD, ranging from early to advanced stages. Traditional approaches often rely on sophisticated AI techniques to analyze functional magnetic resonance imaging (fMRI) data from the patient's brain. Nevertheless, the use of fMRI is limited in detecting PD in its early stages as it primarily focuses on imaging the brain, while the primary causative factor of PD, α‐Syn, has been found to originate from the gut.^[^ ^24^ ^]^ We propose a novel convolutional neural network (CNN) based on AlexNet model^[^ ^25^ ^]^ for detecting the stages of PD using images obtained from gut‐microenvironment‐triggered imaging sensors. The proposed method first utilizes fluorescence images of the abdomen of PD mice to quantify the concentration of α‐Syn, and then extracts relevant features to predict the stages of PD. To verify the learning performance of the trained CNN model, we compared it with five benchmark machine learning algorithms, including Tree,^[^ ^26^ ^]^ support vector machines (SVM),^[^ ^27^ ^]^ K‐nearest neighbor (KNN),^[^ ^28^ ^]^ linear discriminant analysis (LDA),^[^ ^29^ ^]^ and naive Bayesian (NB).^[^ ^30^ ^]^ The experimental results show that the proposed method outperforms the benchmark methods in all metrics, achieving a testing accuracy (correct prediction rate of PD stages over the testing dataset) higher than 98%.

This research represents a significant step forward in the field of neurodegenerative disease diagnosis and treatment. By incorporating AI with cutting‐edge imaging technology and the study of gut microenvironment, we have developed a powerful tool for predicting PD with high accuracy. Our approach offers two notable advantages. First, the development of an originality imaging sensor (Eu‐MOF@Au‐Aptamer) enables the detection of α‐Syn changes in the gut microenvironment associated with PD, providing a non‐invasive approach for PD diagnosis. Second, the integration of AI allows for the analysis of sensor data and the creation of a predictive model, enabling accurate and early prediction of PD. These findings have significant implications for early detection, treatment, and understanding of the gut‐brain axis in PD.

## 2 Results

### 2.1 Characterization of Gut‐Microenvironment‐Triggered Imaging Sensors

**Figure 2:**
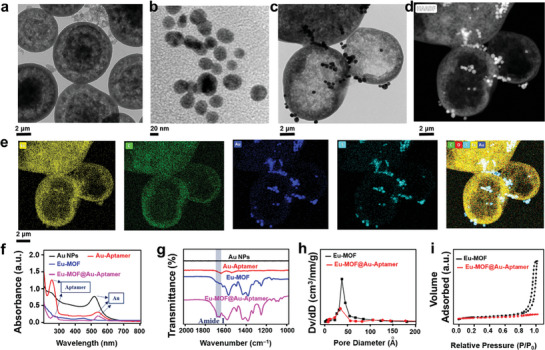
Characterization of Eu‐MOF, Au‐Aptamer, and imaging sensor. a) TEM micrograph of luminescent Eu‐MOF hollow spheres, b) Au nanoparticle, and c) gut‐microenvironment‐triggered imaging sensor (Eu‐MOF@Au‐Aptamer), d) aberration‐corrected high‐angle dark‐field scanning transmission electron microscopy (STEM) image of Eu‐MOF@Au‐Aptamer and e) nanoscale element mappings; we obtained the UV–vis f) and FT‐IR g) spectra of suspensions of a gut‐microenvironment‐triggered imaging sensor and its components (Au nanoparticle, Au‐Aptamer, and Eu‐MOF); distribution of pore sizes h) and *N*_2_ adsorption‐desorption isotherms i) in Eu‐MOF (before) and gut‐microenvironment‐triggered imaging sensor (after).

The micron‐level hollow luminescent MOF was produced at 180 °C to prevent the luminescent Eu‐MOF particles from being absorbed by intestinal epithelial cells and transferred into the body following oral administration. The particles that can be absorbed by enterocytes typically have diameters ranging from 50 nm to 500 nm.^[^ ^31^ ^]^ **Figure** **2**a and Figure S1a (Supporting Information) show that the Eu‐MOF has a virtually hollow sphere structure, with a diameter of 6 microns. The hollow form of the Eu‐MOF was depicted by the dynamic light scattering (DLS, *n* = 6 independent experiments), which has a zeta potential of 25.1±4.2 mV (Figure S2, Supporting Information) and an average diameter of 7.3±1.7 µm by dynamic light scattering (DLS).

To validate the successful loading of the aptamer into the luminescent Eu‐MOF particles with a defined pore size, a series of characterization techniques were employed, including transmission electron microscopy (TEM), Brunauer‐Emmett‐Teller (BET) spectroscopy, Fourier‐transform infrared (FT‐IR) spectroscopy, and ultraviolet‐visible spectroscopy (UV–vis). These methods were used to provide proof of the successful loading. Through TEM, spherical gold nanoparticles are shown in Figure 2b. The fact that the surface of the Au‐Aptamer is exactly loaded into the mesoporous luminescent Eu‐MOF via the pore can be seen in Figure 2c–e and Figure S1b (Supporting Information) as well as in other figures. Meanwhile, the results of the nanoscale mapping (Figure 2e) demonstrate the successful synthesis of the gut‐microenvironment‐triggered imaging sensor (Eu‐MOF@Au‐Aptamer). Additionally, Figure S3 (Supporting Information) contrasts the as‐prepared Eu‐MOFs to the simulated Eu‐MOFs (crystallographic data center number: 1023321),^[^ ^32^ ^]^ and the findings are identical to simulated powder X‐ray diffraction (XRD) data (Figure S3, Supporting Information). Figure 2f shows the UV–vis absorption spectra of the gut‐microenvironment‐triggered imaging sensor (Eu‐MOF@Au‐Aptamer) and its constituent components (Au nanoparticles (Au NPs), Aptamer, Au‐Aptamer, and Eu‐MOF) in the UV–vis spectrum. The pink curve (gut‐microenvironment‐triggered imaging sensor) represents the particular absorption peak of aptamer at 265 nm and the peak absorbance of Au NPs at 530 nm.^[^ ^33^ ^]^ Eu‐MOF@Au‐Aptamer particles show typical peaks of aptamer in the 1640–1660 cm^−1^ (amide I band) region in the FT–IR spectra, showing that aptamer has been effectively encapsulated on the Eu‐MOF particles^[^ ^34^ ^]^ (Figure 2g). The data presented above indicate the successful encapsulation of Au‐Aptamer into the Eu‐MOF.

To further validate the capability of tailoring the pore size of Eu‐MOF to interact favorably with the Au‐aptamer, the BET method was employed to assess the pore size distributions and nitrogen adsorption‐desorption isotherms of both mesoporous Eu‐MOF@Au‐Aptamer particles and bare Eu‐MOF particles.^[^ ^35^ ^]^ The N_2_ adsorption‐desorption isotherm, as illustrated in Figure 2h, demonstrates that the gut‐microenvironment‐triggered imaging sensor has a reduced surface area compared to the parent Eu‐MOF, which is consistent with previous observations. It is also noticeable that when the density function theory (DFT) technique is used, the mesopore volume and pore volume are significantly reduced in the pore size distribution (Figure 2i).^[^ ^36^ ^]^ These results indicate that the intended pore size of Eu‐MOF capsules can efficiently load Au‐Aptamer. The results of the zeta potential tests, depicted in Figure S4 (Supporting Information), indicate that the Eu‐MOF particles exhibit a positive charge. The addition of the negatively charged gut‐microenvironment‐triggered imaging sensor results in a shift in the potential from 25.1 to −7.4 millivolts, implying the successful incorporation of the aptamer into the pores and modification of the surface charge and pore size.

### 2.2 Cytotoxicity of the Gut‐Microenvironment‐Triggered Imaging Sensor

**Figure 3:**
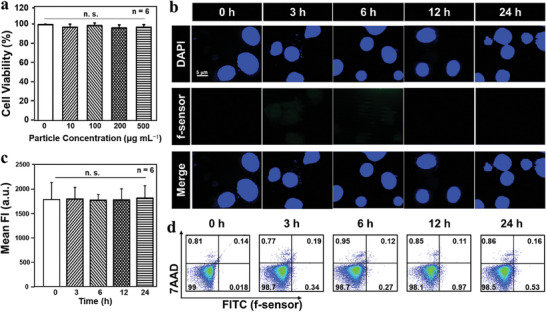
Cytotoxicity and internalization of the imaging sensor. a) Cytotoxicity of a gut‐microenvironment‐triggered imaging sensor at various concentrations at suspensions on Caco‐2 cell monolayers. Images obtained utilizing confocal laser scanning microscopy (CLSM) in b) and flow‐cytometry analysis in c) and d) of Caco‐2 cell monolayers that had been treated with a fluorescence‐labeled gut‐microenvironment‐triggered imaging sensor for a preset amount of time. The term “MFI” in c) refers to the “mean fluorescence intensity”, and 7‐Amino‐Actinomycin D (7‐AAD) in d), is a viability probe used for methods of exclusion of dead cells. n.s. stands for “not significant”, and *n*  =  6 in a) and c) means that the experiment was repeated six times.

The suitability of the human colon epithelial Caco‐2 cell line as an in vitro model for evaluating the cytotoxicity of the gut‐microenvironment‐triggered imaging sensor was determined through dose‐dependent assessments. The Caco‐2 cell line, known for its utility in investigating intestinal absorption, has been utilized in previous studies and was deemed appropriate for the current investigation.^[^ ^37^ ^]^ A comparison of the viability of fluorescein isothiocyanate (FITC) labelled gut‐microenvironment‐triggered imaging sensor (f‐sensor)‐treated and untreated cells is shown in **Figure** **3**a, demonstrating that the gut‐microenvironment‐triggered imaging sensor is not toxic, where *P* value greater than 0.05 (*P>*0.05) was considered not statistically significant.

### 2.3 Caco‐2 Uptake of a Microenvironment‐Triggered Imaging Sensor

To ensure accurate detection of α‐Syn in the gut, it is imperative to prevent the gut‐microenvironment‐triggered imaging sensor from being absorbed by intestinal epithelial cells. Cell uptake was evaluated by incubating Caco‐2 cells with a fluorescence‐labeled gut‐microenvironment‐triggered imaging sensor at 37 °C, followed by the analysis of uptake using CLSM. The internalization rate is measured using flow cytometry with the fluorescence‐labeled gut‐microenvironment‐triggered imaging sensor. As shown in Figure 3b–d, the investigation revealed that there was no substantial cellular uptake of the gut‐microenvironment‐triggered imaging sensor, with a diameter of ≈7 µm, over the course of time. Numerous studies have revealed that Caco‐2 cells are not capable of absorbing particles with a size of one micron or larger.^[^ ^38^ ^]^ As shown in Figure 3a, it is confirmed that the particles do not exhibit toxicity to Caco‐2 cells.

### 2.4 Stability of Gut‐Microenvironment‐Triggered Imaging Sensor Under GI Conditions

**Figure 4:**
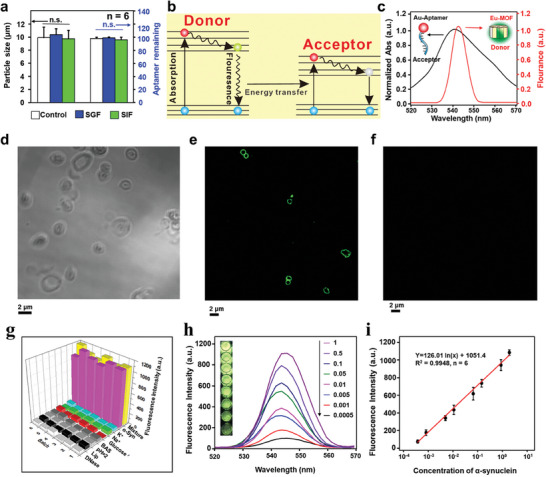
Mechanism test of the image sensor. a) Comparison of particle size and aptamer content in the imaging sensor before (Control) and after treatment with simulated gastric fluid (SGF) and simulated intestinal fluid (SIF) (n  =  6 independent experiments). b) diagram illustrating the FRET process. c) Fluorescence emission of bare Eu‐MOF and UV–vis absorption spectra of Au‐Aptamer NPs. d) Confocal laser scanning microscopy (CLSM) images of bare Eu‐MOF, with a bright field image, e) a fluorescence image, and f) CLSM images of the gut‐microenvironment‐triggered imaging sensor after Eu‐MOF's fluorescence intensity suppression. g) Specificity of the proposed gut‐microenvironment‐activated imaging sensor within the mixture containing DNase, lipid, pH = 2, BSA, Glucose, Na^+^, K^+^, and α‐Syn. h) Spectra of fluorescence generated by the “turn‐on” strategy for fluorescence generation using a variety of concentrations of α‐Syn, where the insets depict the visualization of the diagram with different concentrations of α‐Syn. i) A standard curve depicts the relationship between the fluorescence intensity and different concentrations of α‐Syn measured at 545 nm, where emission intensities of luminescent Eu‐MOF found in aqueous solutions were measured at 450 nm.

A gut‐microenvironment‐triggered imaging sensor needs to traverse the gastrointestinal tract, which is regarded as the most challenging environment in the body before it can specifically target α‐Syn and generate a fluorescence signal in the gut.^[^ ^39^ ^]^ The as‐optimized gut‐microenvironment‐triggered imaging sensors were put in 37 °C simulated gastric fluid (SGF, pH 2.0) and intestinal fluid (SIF, pH 7.0) to evaluate their stability in the GI environment.^[^ ^40^ ^]^ **Figure** **4**a indicates that the particle size and aptamer remained in the tested gut‐microenvironment‐triggered imaging sensor treated in SGF or SIF remained equivalent to those in the untreated control counterparts. This research reveals the physical stability of gut‐microenvironment‐triggered imaging sensors under the circumstances in the GI tract.

To further affirm the chemical and optical robustness of the developed sensor, we employed Fourier transform infrared spectroscopy (FT‐IR) and fluorescence spectrophotometry to assess its performance under simulated gastrointestinal fluid conditions. As depicted in Figure S5a (Supporting Information), the nanoparticles unequivocally exhibit remarkable chemical stability under simulated gastrointestinal tract conditions. Subsequently, we investigated the fluorescence behavior of the nanoparticles using a molecular fluorometer (Figure S5b, Supporting Information). Remarkably, while minimal fluorescence was detected in the simulated gastrointestinal fluid, it is imperative to note the absence of α‐Syn in this fluid. Intriguingly, upon the introduction of solutions containing α‐Syn designed to mimic gastric and intestinal fluids, fluorescence became evident in the gut‐microenvironment‐triggered imaging sensor solutions. This observation underscores the unwavering optical performance of our nanoparticles even within the dynamic environment of the gastrointestinal tract.

### 2.5 Gut‐Microenvironment‐Triggered Imaging Sensor Measurement Mechanism

To enable effective target detection, we proposed a fluorescence “turn‐on” strategy that relied on the quenching of the luminescent europium‐based Eu‐MOF by the Au‐aptamer nanoparticle through FRET (Figure 4b). The absorbance of the Au‐aptamer nanoparticle was measured using UV–vis spectrophotometry to confirm the potential for energy transfer. As shown in Figure 4c (black curve), the unique absorbance of the Au‐aptamer nanoparticles was observed in the wavelength range of 520–570 nm, with the maximum absorbance at 540 nm. Concurrently, it is found that the luminescent Eu‐MOF has a fluorescence emission range of 520–570 nm with a peak at 545 nm (Figure 4c, red curve). The substantial spectral overlap between the fluorescence emission of the Eu‐MOF and the surface plasmon absorption of the Au‐aptamer nanoparticle allowed for efficient energy transfer through fluorescence resonance energy transfer in this system.

As depicted in Figure 4d,e, the bare Eu‐MOF is able to exhibit intense fluorescence upon exposure to light (CLSM images). However, after the production of the gut‐microenvironment‐triggered imaging sensor, the presence of the Au nanoparticles effectively suppressed the fluorescence intensity of the Eu‐MOF (Figure 4f).^[^ ^41^ ^]^ This research has demonstrated the ability of Au‐aptamer nanoparticles (NPs) to effectively quench the luminescent light emission of the Eu‐MOF. These results support the feasibility of utilizing a fluorescence “turn‐on” strategy for the non‐invasive monitoring of α‐Syn by oral administration. Overall, these findings suggest that the gut‐microenvironment‐triggered imaging sensor can potentially be a valuable tool for the early diagnosis of PD.

### 2.6 Selectivity of the Gut‐Microenvironment‐Triggered Imaging Sensor

To assess the selectivity of gut‐microenvironment‐triggered imaging sensor by oral delivery, primary interferences included in the GI tract (DNase, lipid, pH = 2, bovine serum albumin (BSA), Glucose, Na^+^, and K^+^) were utilized.^[^ ^42^ ^]^ Specifically, each of these potential interferences was independently added to bile extract, which was used to mimic the gut microenvironment.^[^ ^8^ ^]^ A comparison of the fluorescence intensity obtained with and without introducing the above‐mentioned interferences is presented in Figure 4g. The results demonstrate that the presence of the interferences had no significant effect on the fluorescence intensity of the gut‐microenvironment‐triggered imaging sensor. Turning on fluorescence can successfully distinguish α‐Syn from other proteins, and the main components of the gut do not exhibit significant cross‐reactivity in the gastrointestinal tract.

### 2.7 Properties of the Gut‐Microenvironment‐Triggered Imaging Sensor

A fluorescence “turn‐on” technique based on FRET is utilized to accomplish the quantitative detection of α‐Syn in the GI environment. In Figure 4h, it can be observed that a linear relationship exists between the fluorescence intensity measured at 545 nm and the concentration of α‐Syn when the target concentration is between 0.0005 and 1 ng mL^−1^. The color of fluorescence can be observed with the naked eye at different concentrations of α‐Syn solution. The relationship between the fluorescence intensity and the concentration of α‐Syn is well‐described by the equation y = 126.01 ln(x) + 1051.4, with a high correlation coefficient of 0.9948 (Figure 4i). When the signal‐to‐noise ratio (S/N) equals 3, the minimum amount of antigen that can be detected is predicted to be 0.1 pg mL^−1^. The repeatability of the fluorescence “turn‐on” strategy is evaluated based on the results of six separate detections performed at concentrations of 1 ng mL^−1^ and 0.5 pg mL^−1^ α‐Syn. The data exhibit a remarkable 2.89% and 1.87% relative standard deviation (RSD). The presented non‐invasive intestinal detection PD strategy was successfully employed to measure a practical sample of a wide range of α‐Syn concentrations, as demonstrated above. **Table** **1** lists many strategies for detecting α‐Syn compared to the development methodology. Results show that the fluorescence “turn‐on” method can be utilized to analyze a wide range of concentrations of α‐Syn in a practical sample using a fluorescence detector. Therefore, it has been demonstrated that the fluorescence “turn‐on” strategy provides a non‐invasive method for diagnosing PD.

**Table 1:** Comparison of this work with reported assays for detecting α‐Syn.

Detection method	Detection range	Limit of detection (LOD)	Approach	In vivo	Predictive function	Real‐time monitoring	Ref.
Electrochemical immunosensor	10–1000 ng mL^−1^	1.13 ng mL^−1^	Invasive	No	No	No	[43]
Surface plasmon resonance (SPR)	0–70 000 ng mL^−1^	112 pg mL^−1^	Invasive	No	No	No	[44]
EIS	0.014–196 ng mL^−1^	7 pg mL^−1^	Invasive	No	No	No	[45]
Electrochemical	1–1000 ng mL^−1^	310 pg mL^−1^	Invasive	No	No	No	[46]
Localized surface plasmon resonance (LSPR)	980–9800 ng mL^−1^	980 000 pg mL^−1^	Invasive	No	No	No	[47]
Fluorescent probes	672–4942 ng mL^−1^	560 pg mL^−1^	Invasive	No	No	No	[48]
Artificial Intelligence‐Guided Gut‐Microenvironment‐Triggered Imaging Sensor	0.0005−1 ng mL^−1^	0.04 pg mL^−1^	Non‐invasive	Yes	Yes	Yes	This work

### 2.8 In Vivo Detection α‐Syn

In this study, C57BL/6 mice with 1‐Methyl‐4‐phenyl‐1,2,3,6‐tetrahydropyridine (MPTP)‐induced neurodegeneration were used as a PD model, in which the presence of pronounced α‐Syn in the gut was observed. To comprehensively assess the stability of the MPTP‐induced PD model, we conducted post‐modeling behavioral experiments at various time intervals. Notably, meticulous observations of mouse behavior within the initial two‐month period revealed pronounced PD characteristics (refer to Figure S6, Supporting Information). Concurrently, we scrutinized the intestinal synaptic proteins of mice aged two months, confirming their compliance with our testing criteria throughout this duration. The allocated two‐month timeframe proved ample for the complete execution of all experiments, thereby affirming the model's applicability in accordance with our rigorous experimental standards.

**Figure 5:**
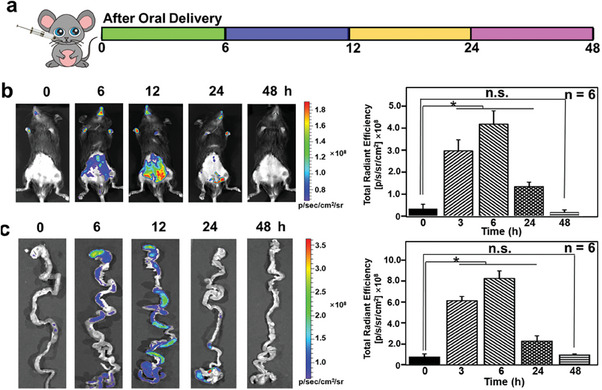
Imaging sensor precision detection of PD. a) A schematic depiction of the time course of monitoring mice after oral drug administration. b) Images obtained from an in vivo imaging system (IVIS) depicting the biodistribution of a gut‐microenvironment‐triggered imaging sensor after test mice were given the imaging sensor orally. c) Images obtained using ex vivo IVIS depicting the accumulation of luminous Eu‐MOF and their related total radiant efficiencies from the isolated guts of the test mice after they had been harvested, where n.s. stands for “not significant”; * denotes *p*<0.05, which was considered statistically significant.

The viability of utilizing a gut‐microenvironment‐triggered imaging sensor to monitor PD via the gastrointestinal system and feces noninvasively was examined, from proof of concept to practical implementation (**Figure** **5**a).^[^ ^35^ ^]^ The fluorescence intensity of luminescent Eu‐MOF begins to emerge from the abdomen ≈6 h after oral administration of an imaging sensor triggered by the gut microenvironment. This signal dramatically increases over time and reaches a plateau 12 h after oral administration of the imaging sensor. At 48 h, the fluorescence intensity gradually decreases in the abdomen and as the feces are eliminated from the system. This continues for the next 24 h (Figure 5b). Therefore, for the subsequent AI data processing, we selected 12 h as the time point for collecting mouse abdominal data. This study revealed that the non‐invasive intestinal detection PD strategy can be utilized to monitor α‐Syn in the gastrointestinal tract in a non‐invasive manner. After oral delivery of gut‐microenvironment‐triggered imaging sensors, ex vivo data demonstrate that the fluorescence signals of luminescent Eu‐MOF were primarily observed existent in the stomach (Figure 5c). The fluorescence signal travels from the stomach to the intestine and finally to the colon, commencing in the stomach (Figure 5c). The assessment of luminous Eu‐MOF fluorescence intensity using the region of interest (ROI) method illustrates the kinetics of the transit of gut‐microenvironment‐triggered imaging sensors throughout distinct segments of the gastrointestinal tract (Figure 5c). According to inductively coupled plasma mass spectrometry (ICP‐MS), 98.38 percent (Figure S7, Supporting Information, *n* = 6) of the orally administered gut‐microenvironment‐triggered imaging sensors are detected in the feces 48 h after the gavage, indicating that the majority of the particles are excreted through feces, with only a minimal fraction being absorbed into the body.

Using an oral delivery gut‐microenvironment‐triggered imaging sensor, the level of α‐Syn in the feces of PD mice was analyzed after 48 h to evaluate the reliability and potential clinical applications of this method. The accuracy of α‐Syn detection in fecal samples was determined by examining the recovery of α‐Syn from fecal samples using a conventional approach. The results of α‐Syn detection in feces were used to establish a novel approach, which was then compared to a commercially available ELISA kit. The proposed non‐invasive gut‐based detection of PD using fluorescence was validated through comparison with the results of an ELISA kit experiment, demonstrating the high accuracy of the proposed method for α‐Syn detection (**Table** **2**).

**Table 2:** Comparison of a non‐invasive intestinal PD detection strategy with a commercially available ELISA Kit (x¯ ± s*, n* = 6).

Sample	Background (µg mL^−1^)	Added Concentration	ELISA (µg mL^−1^)	Developed method	Recovery (%)
1	2.000	0.050	2.059±0.011	2.051±0.001	102.00
2	0.400	0.100	0.495±0.011	0.498±0.009	98.00
3	0.000	0.500	0.490±0.050	0.493±0.091	98.60
4	0.000	1.000	0.980±0.040	0.982±0.060	98.20
5	0.300	5.000	5.310±0.250	5.310±0.150	100.20
6	5.000	10 000	14.508±0.312	14.489±0.249	94.89

Some unique attributes of the imaging sensor for the analysis of α‐Syn are as follows. a) Non‐invasive gut testing: Gut‐triggered sensors detect the origin of Parkinson's Disease in the gut, whereas ELISA is unsuitable for intestinal detection. b) Sensitivity and specificity: While ELISA kits can detect α‐Syn in feces, the imaging sensor offers superior sensitivity (LOD 0.04 pg mL^−1^) and specificity. ELISA has a detection limit (7.2 pg mL^−1^) but is unable to accurately detect low levels of α‐Syn, particularly in the early stages of Parkinson's Disease. In contrast, the imaging sensor provides enhanced sensitivity, enabling real‐time visualization of even trace amounts of α‐Syn, which is crucial for early diagnosis and disease progression monitoring. c) Localization and spatial information: ELISA kits provide information regarding the presence of α‐Syn but lack spatial information. In contrast, the imaging sensor enables in vivo sensing, offering precise localization and spatial distribution of α‐Syn in the gut. This is critical for understanding the specific regions affected by α‐Syn pathology and investigating aggregation patterns and their implications for disease progression. d) Real‐time monitoring and dynamic analysis: ELISA is a static measurement that provides information at a single time point. In vivo sensing with the imaging sensor allows for real‐time monitoring of α‐Syn levels and dynamics within the gut. By continuously imaging the gut, researchers can observe changes in α‐Syn over time, track disease progression, and evaluate the efficacy of therapeutic interventions. This dynamic analysis provides deeper insights into the temporal aspects of α‐Syn pathology. e) Potential for multimodal analysis: The imaging sensor can be combined with other imaging modalities, such as non‐invasive molecular imaging or functional imaging techniques, to obtain a more comprehensive understanding of α‐Syn in the gut. Integration of different imaging modalities allows researchers to correlate α‐Syn levels with other biomarkers, changes in the gut microenvironment, or functional alterations, for a more holistic analysis of PD. f) Future applications and personalized medicine: The imaging sensor's ability to provide real‐time, non‐invasive monitoring of α‐Syn in the gut opens up possibilities for personalized medicine and tailored treatment approaches. By understanding an individual's α‐Syn profile, clinicians can customize therapies and interventions, monitor treatment response, and potentially intervene before irreversible damage occurs.

In summary, apart from the utility as ELISA, the gut‐microenvironment‐triggered imaging sensor can prospectively upgrade and/or improve monitoring, diagnostics, and treatment to the PD thanks to its unique attributes mentioned above.

To gain a deeper understanding of the non‐invasive monitor α‐Syn based on gut‐microenvironment‐triggered imaging sensors, CLSM images are used to examine the gut analysis of orally administered gut‐microenvironment‐triggered imaging sensor in healthy mice and PD mice. Fluorescence signals of luminescent Eu‐MOF have manifested in PD mice, as evidenced by CLSM pictures taken ex vivo (Figure S8, Supporting Information). It is noteworthy that the fluorescent signals generated by the gut‐microenvironment‐triggered imaging sensor were not observed in the intestines of healthy mice. This is due to the absence of pathogenic α‐syn alterations in the gastrointestinal system of these mice, which precludes the initiation of the fluorescence switch strategy. Therefore, studies have demonstrated that this non‐invasive approach to detect α‐syn in the gut can be used to diagnose PD.

### 2.9 In Vivo Toxicity

To assess the safety of the oral gut‐microenvironment‐triggered imaging sensor in vivo, histological sections of the gastrointestinal tract from treated mice were evaluated for evidence of toxicity at the endpoint. Figure S9 (Supporting Information) shows that there is no apparent inflammatory response in the experimental tissues, suggesting no significant in vivo toxicity. This is largely due to the fact that the imaging sensors activated by the gut microenvironment are not absorbed when administered orally.

### 2.10 Classification of Mice PD Models

To effectively develop AI methods for predicting the various stages of Parkinson's disease in mice, it is essential to first identify these different stages accurately. To achieve this, we conducted a comprehensive assessment of the animals' disease progression through various animal behavior experiments, including the open field test, rotarod test, and water maze test. The open field test, and water maze test are commonly used behavioral testing methods to assess different stages of PD in mice.^[^ ^49^ ^]^ The open field test can be used to assess changes in motor ability and coordination in mice at different stages of PD.^[^ ^50^ ^]^ The rotarod test is commonly used to assess motor coordination and balance, making it a valuable tool in evaluating motor defects in PD.^[^ ^51^ ^]^ The water maze test can be used to detect changes in learning and memory function in mice at different stages of PD.

**Figure 6:**
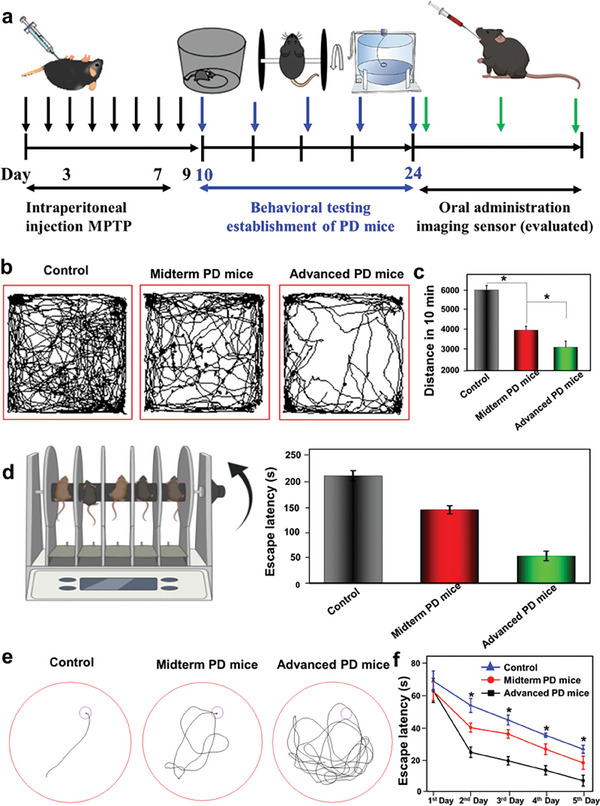
A schematic diagram illustrating the experimental setup for determining different stages of PD. a) The experiment trajectory, b) travel distance, and c) observed distance in 10 min during the open field test in mice from different groups; d) rotarod test of PD mice, and escape trajectory; and e) trajectory and f) escape latency recorded during the Morris water maze test in mice from different groups, where statistical analysis indicated significance (*p<0.05) compared to those of the normal (Control) mice and the sample size for each group was *n* = 8‐10.

**Figure** **6**a depicts a schematic diagram illustrating the establishment and determination of the model at different stages of PD. Specifically, control PD mice denote those treated with MPTP for 3 days, whereas midterm PD mice are those treated for 7 days, and advanced PD mice denote those treated for 9 days. The locomotor activity of three groups (Control mice, Midterm PD mice, and Advanced PD mice) was assessed using the Open field test. In Figure 6b, it can be observed that the distance traveled in 10 min on the advanced PD mice decreased to 2852.2 cm (Figure 6b, *p*<0.05). The distance continued to decrease following MPTP treatment (Figure 6c, *p*<0.05). Additionally, the rotarod test, which evaluates motor behavior, revealed a significant 74.88% decrease in latency time for the advanced PD mice compared to the control group, indicating impaired motor function (Figure 6d). To delve deeper into the impact of varying MPTP administration durations on cognition, we employed the Morris water maze (Figure 6e). The escape latency of the three cohorts of mice was gauged: the control group (early PD), mid‐term PD, and late PD. Remarkably, mice in the late PD group exhibited a significantly prolonged escape latency compared to the control group (Figure 6f, *P*<0.05). Notably, the control group displayed the shortest swimming path within the platform area compared to the late PD mice. Meanwhile, the mid‐term PD mice showcased a swimming path length longer than the early PD group but not as extensive as the late PD cohort. These findings underscore the successful establishment of PD mouse models at distinct stages.

### 2.11 Proposed Model

**Figure 7:**
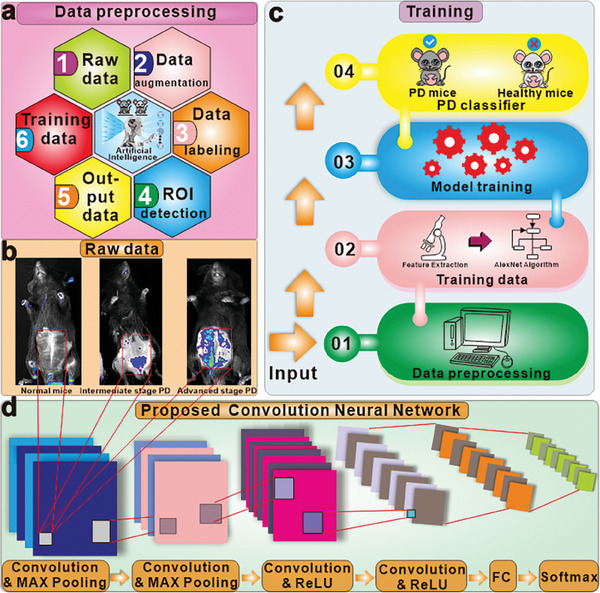
Training framework of the proposed CNN model. a) The data preprocessing, comprising raw data labeling of three stages of PD mice, data augmentation, and ROI detection for extracting the essential information of the raw fluorescence intensity data that are illustrated in b). c) The training process of the proposed model utilizing the augmented dataset and d) the architecture of the proposed CNN model.

To assess and predict PD progression, a customized CNN based on AlexNet is proposed for analyzing and forecasting PD's progression through its various stages as shown in **Figure** **7**, where the data preprocessing (Figure 7a) consists of data labeling, ROI detection and data augmentation; the raw data for three stages (Figure 7b) are input to the CNN training process (Figure 7c); the structure of the obtained CNN (Figure 7d) is also a revised version of AlexNet model with one CNN layer and one max pooling layer removed for reducing the model complexity and the risk of over fitting, and improving the learning performance of the trained CNN model with the limited training data.

### 2.12 Performance Comparison with Baseline Machine Learning Models

To verify the learning performance of the proposed CNN model (denoted by CNN), we compared it with five benchmark algorithms, including Tree, SVM, KNN, LDA, and NB since they are commonly used in machine learning and shown effective in various classification tasks. To evaluate the performance of the proposed algorithm, we use two metrics in the training stage: F1 score, area under the curve (AUC) of the receiver operating characteristic (ROC) curve, and the larger of F1 score and the AUC value, the better the training performance, in addition to the testing accuracy for testing stage. It should be emphasized that these metrics are only applicable to binary classification tasks. To overcome this limitation, we selected one label as the main class and treated the remaining labels as the other class, thereby allowing us to evaluate the proposed algorithm using the above‐mentioned metrics. Moreover, in the preformed mice experiments, the early stage or normal, the middle stage, and the advanced stage of PD are labeled as “0”, “1” and “2”, respectively. All models were trained using pre‐processed fluorescence intensity images and tested on a separate set of data to predict the labels of the test samples. The evaluation results were then used to compare the performances of the proposed algorithm and the benchmark algorithms.

**Figure 8:**
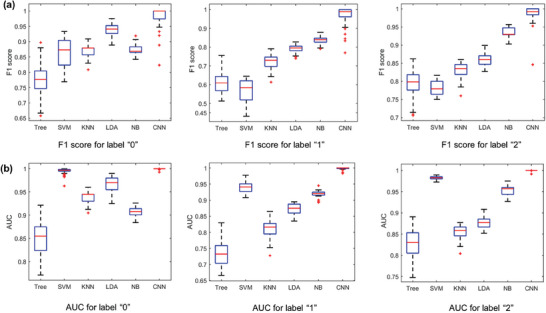
Performance comparisons of the proposed algorithm (denoted by CNN) with benchmark algorithms (Tree, SVM, KNN, LDA, NB). a) F1 score comparison. b) AUC comparison.

**Figure** **8**, Figures S10 and S11 (Supporting Information) present a performance comparison of the proposed algorithm with some benchmark algorithms, demonstrating that the proposed algorithm significantly outperforms the other algorithms in all metrics, achieving a testing accuracy higher than 98% in all experiments. Moreover, the proposed revised AlexNet model exhibits consistent best performance across all data labels.

## 3 Discussions

First of all, the development of an AI‐Guided gut microenvironment triggered Imaging Sensor for Precision Prediction of PD is a significant breakthrough in the field of healthcare technology. The research demonstrates the potential of combining artificial intelligence and advanced imaging sensors to identify and predict the onset of complex diseases. This technology could effectively realize earlier diagnosis and more personalized treatment plans for patients with PD, thereby improving their quality of life.

Additionally, the gut microenvironment plays a crucial role in PD pathogenesis. The sensor developed in this study can detect subtle changes in the gut microenvironment that may occur years before clinical symptoms of PD appear. By detecting these changes, the sensor can predict the onset of PD with high accuracy. This research not only contributes to the understanding of PD pathology but also highlights the potential of the gut microbiome as a therapeutic target for the disease.

Lastly, the development of this imaging sensor underscores the importance of interdisciplinary research in healthcare. The study combines expertise from multiple disciplines, including AI, microbiology, and imaging technology. This interdisciplinary approach is essential to stride across complex healthcare challenges. The research not only provides a tool for the early diagnosis of PD but also demonstrates the potential of using AI and advanced sensors to monitor and predict a wide range of diseases and health conditions.

## 4 Conclusion

In this paper, we have presented an AI‐guided imaging sensor, which employs an acid‐resistant, luminescent Eu‐MOF with a precisely defined pore size and Au‐Aptamer nanoparticle complex, for non‐invasive PD monitoring and diagnosis. The presented mice experiments justify the fact that Eu‐MOF can effectively encapsulate and protect the aptamer in harsh gastrointestinal conditions, and that in response to the presence of *α*‐Syn in the gut of the mice, the fluorescence signal of Eu‐MOF will be activated, and its intensity increases with the amount of aptamer/*α*‐Syn complex released. The integration of AI bestows precision upon predictions across various stages of PD, achieving a remarkable accuracy rate exceeding 98%. These findings have demonstrated the potential of the proposed gut‐microenvironment‐triggered imaging sensor combined with the customized CNN model as an effective non‐invasive tool for monitoring PD.

## 5 Experimental Section

### Materials and Reagents

Glucose, 3,3*^′′′^*‐dihydroxy‐2*^′^*,2*^′′^*,5*^′^*,5*^′′^*‐tetramethyl‐ [1,1*^′^*:4*^′^*,1*^′′^*:4*^′′^*,1*^′′′^*‐quaterphenyl]‐4,4*^′′′^*‐dicarboxylic acid, chloroauric acid and europium nitrate were acquired from Alfa Aesar (Tewksbury, MA, USA). Sangon Biotech (Shanghai, China; https://www.sangon.com) produced the *α*‐Syn aptamer sequence of 5*^′^*‐SH‐TTTTTGGTGGCTGGAGG GGGGGCGC‐GAACG).^[^ ^52^ ^]^ Sigma–Aldrich supplied the liquid, pepsin, deoxyribonuclease (DNase), and bile extracts for this study (St. Louis, MO, USA). Abcam developed the enzyme‐linked immunosorbent test (ELISA) kit for the *α*‐Syn ELISA (Cambridge, UK). The additional chemicals that were used were of the analytical grade throughout the entire process.

### Synthesis of Au‐Aptamer Nanoparticle

Gold nanoparticles (AuNPs) were synthesized following a previously reported method53. In brief, a boiling solution of 0.01 wt.% HAuCl4 was vigorously stirred, and 2.5 mL of a 1 wt.% trisodium citrate solution was added to the mixture. The mixture was vigorously stirred, resulting in the formation of AuNPs, as indicated by the gradual change in the color of the solution from gray to blue, purple, and eventually wine red. The solution was maintained at boiling temperature while being stirred vigorously for 10 min to ensure the completion of the reaction. The solution was then allowed to cool to room temperature before being stored at 4 °C until further use.

The synthesis of Au‐Aptamer NPs was accomplished by combining gold and an aptamer. The procedure involved mixing 1 µM of gold and 2 µM of the aptamer and stirring the mixture slowly at 37 °C for 4 h. Following the synthesis of the Au‐aptamer nanoparticles (NPs), they underwent a thorough washing procedure consisting of three consecutive centrifugal separation steps (10 000 rpm, 10 min) to eliminate unbound aptamers. The resulting Au‐Aptamer NPs combination should be stored at 4 °C until used.

### Preparation and Characterization of the Gut‐Microenvironment‐Triggered Imaging Sensor

Solvent thermal synthesis was used to produce luminescent Eu‐MOF.^[^ ^54^ ^]^ 3,3*^′′′^*‐dihydroxy‐2*^′^*,2*^′′^*,5*^′^*,5*^′′^*‐tetramethyl‐[1,1*^′^*:4*^′^*,1*^′′^*:4*^′′^*,1*^′′′^*‐ quaterphenyl]‐4,4*^′′′^*‐dicarboxylic acid and Eu(NO_3_)_3_ (136 mg, 0.3 mmol) was added to a DMF solution that was 10 milliliters in volume and contained 43.5 mg (0.03 mmol) of the compound. The produced solution was loaded into a stainless steel and Teflon autoclave and heated to 180 °C for 12 h. The resulting product was washed with DI water, dried, and stored in a glove box compartment.

To efficiently load Au‐aptamer nanoparticles into luminescent Eu‐MOF, 10 mg of dry luminescent Eu‐MOF was taken out of the glove box and mixed with 10 mL of 50% w/w aptamer for 5 h at room temperature. To get the aptamer off the Eu‐MOF surface, the particle was washed four times with DI water, ethanol, and sodium dodecyl sulfate (SDS, which is known to remove surface‐bound biomacromolecules from the particle successfully).^[^ ^55^ ^]^

The chemical structures of the luminescent Eu‐MOF, Au‐aptamer nanoparticle, and gut‐microenvironment‐triggered imaging sensors were determined using FT‐IR and UV–vis absorbance spectrometry. The FT‐IR analysis was performed using a NicoletTM iSTM50 spectrometer from Thermo Fisher Scientific, while the UV‐Vis absorbance spectrometry was conducted using a Cary 60 instrument from Agilent. The X‐ray diffraction (XRD) study was performed at room temperature using an X'Pert‐Pro MPD diffractometer equipped with a Cu K‐radiation source. DLS (Zetasizer Nano ZSE, Malvern Instruments, Worcestershire, UK) was used to determine the particle's size and zeta potential in DI water, and TEM was used to analyze the particle's shape (JEM‐2100F, JEOL Technics, Tokyo, Japan). The detection was accomplished with an Agilent 7500 ICP‐MS (Agilent, Cheshire, UK).

### Cytotoxicity Study

The cytotoxicity of the gut‐microenvironment‐triggered imaging sensor was evaluated by planting Caco‐2 cells in 96‐well plates with Dulbecco's modified Eagle's medium and allowing them to attach for 24 h. This procedure was implemented to determine whether the imaging sensor was harmful to the cells. Then, the cells were treated with a range of doses of a gut‐microenvironment‐triggered imaging sensor (0–500 mg mL^−1^). After 24 h, the test cells' vitality is determined using a Cell Titer‐Glo Luminescent Cell Viability Assay Kit (Promega, Madison, Wisconsin, USA).

### Uptake of Gut‐Microenvironment‐Triggered Imaging Sensors by Caco‐2 Cells

Caco‐2 cells (1×10^6^ cells mL^−1^) were cultured with a FITC labelled gut‐microenvironment‐triggered imaging sensor (f‐sensor) (500 µg mL^−1^) to determine whether or not they could be endocytosed in vitro. After an initial period of incubation (either 0, 3, 12, or 24 h), the cells were collected, washed extensively in PBS, labeled with DAPI, and then analyzed with a flow cytometer (BD Accuri C6, BD Biosciences, San Jose, CA, USA) and a CLSM (Zeiss LSM980, Carl Zeiss, Jena GmbH, Germany).

### Animal

C57 mice, aged 6–8 weeks and weighing 18–20 grams, were sourced from Ensiweier in Chongqing, China. The study followed the “Guide for the Care and Use of Laboratory Animals” throughout. Approval for all animal experiments was obtained from the Esteemed Ethical Review Committees of Sichuan Provincial People's Hospital and the University of Electronic Science and Technology of China, adhering closely to the rigorous standards outlined in the Sichuan Provincial People's Hospital of Health Guide for the Care and Use of Laboratory Animals. Efforts were made to minimize animal usage and reduce distress.

### Biodistribution of Gut‐Microenvironment‐Triggered Imaging Sensors

After 12 h of fasting, the PD mice given daily i.p. injections of MPTP (0.6 mg, 2 mg mL^−1^) for seven days were orally administered the gut‐microenvironment‐triggered imaging sensor. The biodistributions of the accumulated gut‐microenvironment‐triggered imaging sensors in the abdomen and gut were evaluated at predetermined times (0, 6, 12, 24, and 48 h) following treatment using an in vivo imaging system (IVIS, Xenogen, Alameda, CA, USA).

### Detection of α‐Syn in Fecal at PD Mouse

Eppendorf tubes were used to collect fresh feces with PD mice, which were subsequently frozen at −20 °C to be analyzed later. The fecal samples (10 mg) were dissolved in 10 mL DI water and incubated at room temperature with shaking for 10 min, after which they were washed four times with DI water to remove the residual material supernatant, leaving only the residual material. The collected sample was diluted with 200 µL of DI water and analyzed with an F‐4600 spectrophotometer (Hitachi, Japan) and an ELISA Kit (Abcam, United Kingdom) to determine whether or not *α*‐Syn was present.

### Data Preprocessing and Model Training

The fluorescence intensity images were recorded to measure the different stages of PD in mice, as shown in Figure 7b–d illustrates the data preprocessing process, which included data labeling, capturing the region of interest (ROI) and data augmentation. The data labeling step involved annotating labels to the different stages of raw data. The goal of our proposed algorithm was to correctly and reliably classify different stages of PD after training. However, the data may also incorporate extra information from other parts of the mice, which could potentially degrade the learning performance. To resolve this issue, we artificially capture a fixed size of fluorescence image surrounding the nut of each mouse, which is implemented by selecting a fixed‐size region (400×500) around the abdomen of the mice. Subsequently, data augmentation is performed through various transformations, such as rotation, flipping, and injecting salt noise with small variance, to the raw images. Additionally, we used a method called saliency‐guided data augmentation, which involved mixing patches of salient regions to create new images. Salient regions refer to areas with strong fluorescence intensity.^[^ ^56^, ^57^ ^]^ After data augmentation, all augmented images are resized to 224×224 to meet the input size requirements of our algorithm. The detailed data preprocessing steps are visually depicted in Figure S10 (Supporting Information). Note that before applying data augmentation, the raw dataset comprised 10 samples from normal mice, 9 samples from midterm PD mice, and 16 samples from advanced PD mice. After incorporating data augmentation, the total dataset size increased to 40 samples for normal mice, 40 samples for midterm PD mice, and 60 samples for advanced PD mice. To ensure a robust model, 80% of the augmented data was utilized for model training, while the remaining 20% was reserved for model testing. Here, we would like to highlight that our proposed algorithm is a simplified version of the AlexNet model, which maintains good learning performance even with significantly limited training samples.

### Statistical Analysis

All quantitative data are presented as the mean ± standard deviation (mean ± SD). The student's t‐test with two‐tailed confidence intervals was utilized to compare the two groups using GraphPadPrism 8.0 (GraphPad Software, Inc., CA, USA) and Microcal Origin Pro 8.5.1 (Origin Lab. Corp., Northampton, MA, USA). If *P* value less than 0.05 (*P < 0.05), it is considered statistically significant.

### Study Approval

All animal procedures were approved by Animal Care and Use Committee in Sichuan Provincial People's Hospital and the University of Electronic Science and Technology of China (Ethics license, SPPH‐2023540).

## Conflict of Interest

The authors declare no conflict of interest.

## Author Contributions

Y.L. and Y.‐B.M. conceived and designed the experiments. H.‐X.R. provided key insights for the design of experiments; Y.‐B.M. and H.‐X.R. supervised the project. Y.L., C.‐Y.C. and Y.‐B.M. carried out the experiments. Y.L., C.‐Y.C. and Y.‐B.M. prepared figures and analyzed the data. Y.L. and Y.‐B.M. wrote the manuscript. All authors approved the final version of the manuscript.

## Supporting information

Supporting Information

## Data Availability

The data that support the findings of this study are available from the corresponding author upon reasonable request.
