# Supplementary material for: Artificial Intelligence‐Guided Gut‐Microenvironment‐Triggered Imaging Sensor Reveals Potential Indicators of Parkinson's Disease
Source: Adv Sci (Weinh). 2024 Apr 3;11(23):2307819. doi: 10.1002/advs.202307819 (PMC11187919; doi:10.1002/advs.202307819)
Supplement: Supplementary file 1 — Supporting Information [file ADVS-11-2307819-s001.pdf]

ADVANCED SCIENCE
Open Access

Supporting Information

for *Adv. Sci.*, DOI 10.1002/advs.202307819

Artificial Intelligence-Guided Gut-Microenvironment-Triggered Imaging Sensor Reveals Potential Indicators of Parkinson's Disease

*Yiwei Li, Hong-Xia Ren, Chong-Yung Chi and Yang-Bao Miao**

Supporting Information

Artificial Intelligence-Guided Gut-Microenvironment-Triggered Imaging Sensor Reveals Potential Indicators of Parkinson's Disease

Yiwei Li[a, b], *Hong-Xia Ren*[c], *Chong-Yung Chi*[b], *Yang-Bao Miao*[a,*]

Y. Li and Y. B. Miao

[a] Department of Haematology, Sichuan Academy of Medical Sciences & Sichuan Provincial People's Hospital, School of Medicine of University of Electronic Science and Technology of China, No. 32, West Section 2, First Ring Road, Qingyang District, Chengdu 610000, China.

Y. Li and C. Y. Chi

[b] Institute of Communications Engineering & Department of Electrical Engineering, National Tsing Hua University, Hsinchu 30013, Taiwan.

H. X. Ren

[c] Sichuan Technology & Business College, Chengdu 611800, China.

*Corresponding author: Yang-Bao Miao

Email: miaoyangbao@uestc.edu.cn

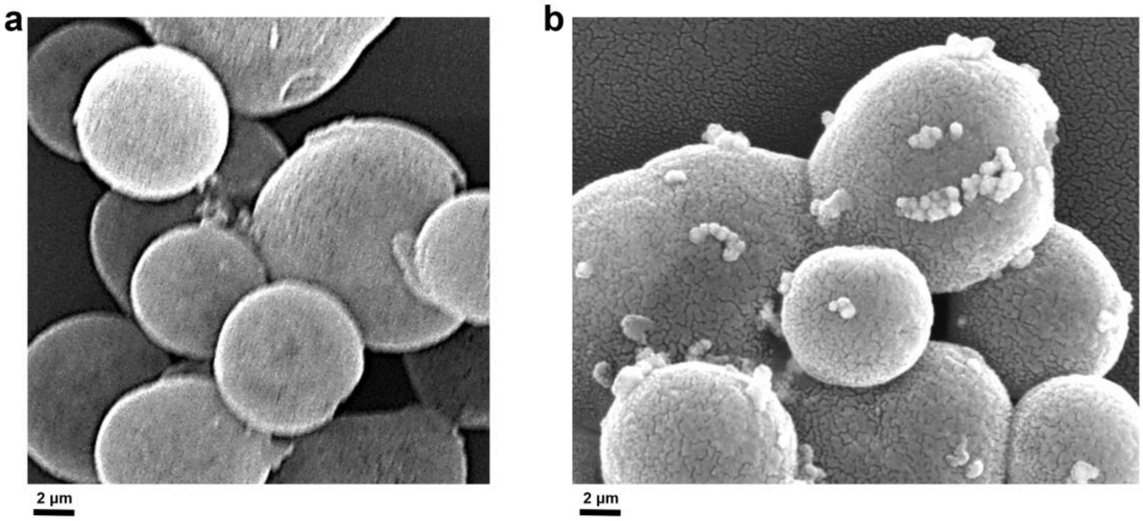

Figure S1. (a) SEM micrograph of luminescent Eu-MOF hollow spheres. (b) gut-microenvironment-riggered imaging sensor (Eu-MOF@Au-Aptamer).

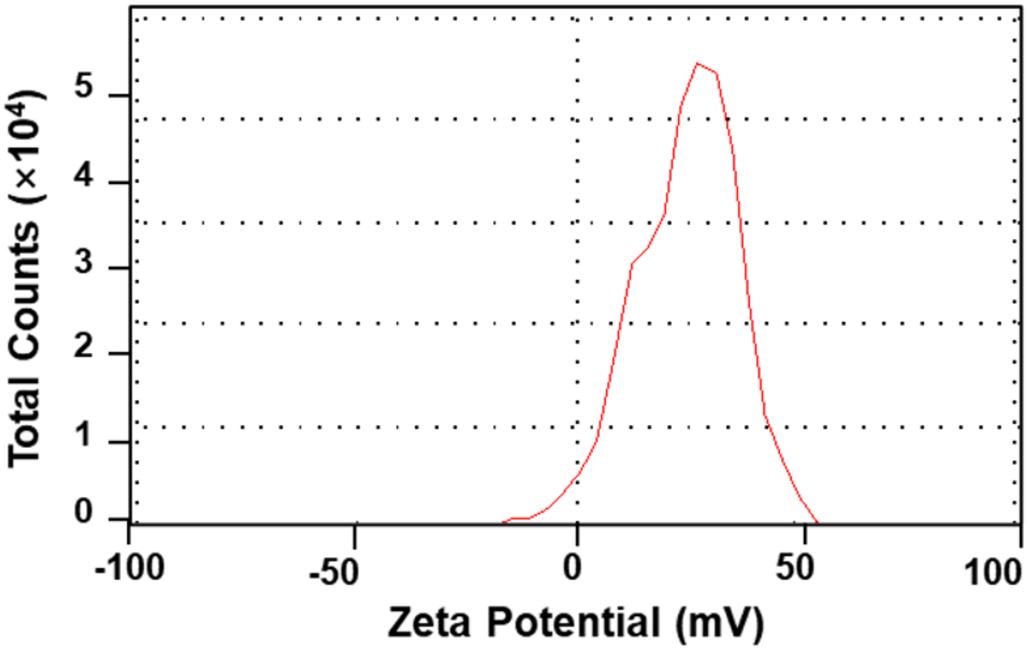

Figure S2. Zeta potential of Eu-MOF analyzed by DLS

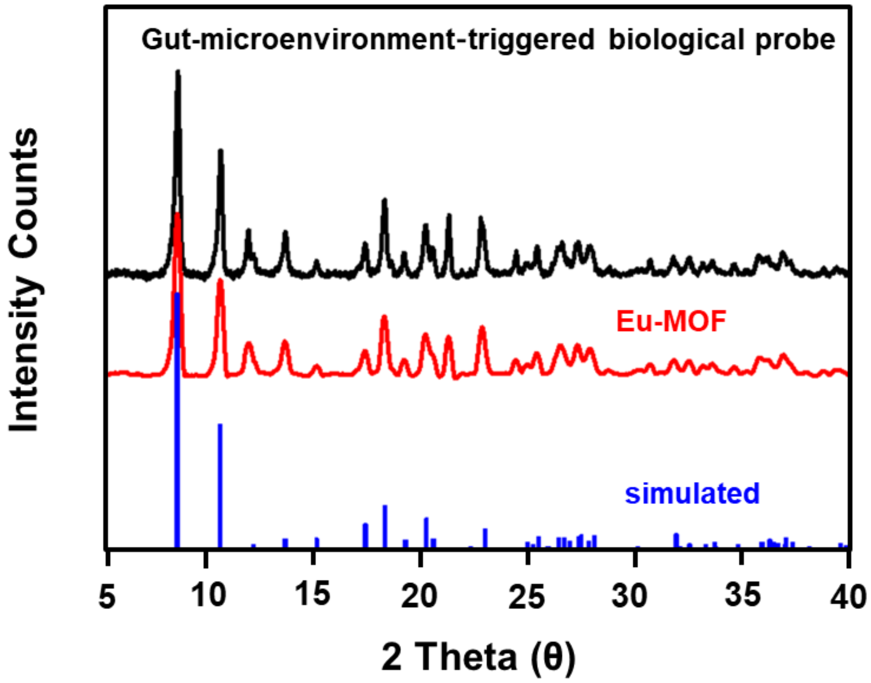

Figure S3. XRD pattern comparison of gut-microenvironment-triggered imaging probe with simulated and as-synthesized Eu-MOF.

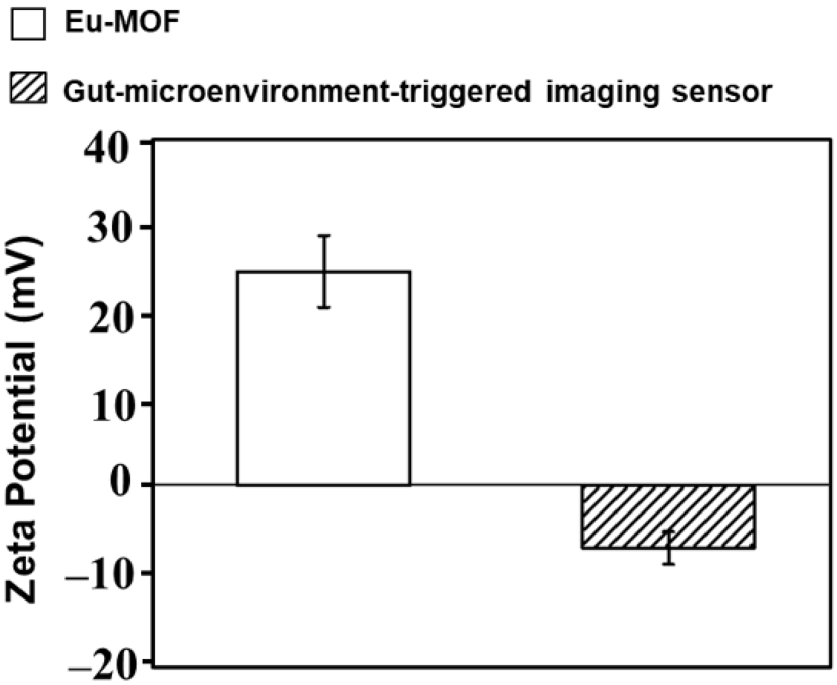

Figure S4. Zeta potential of Eu-MOF and gut-microenvironment-triggered imaging sensor analyzed by DLS.

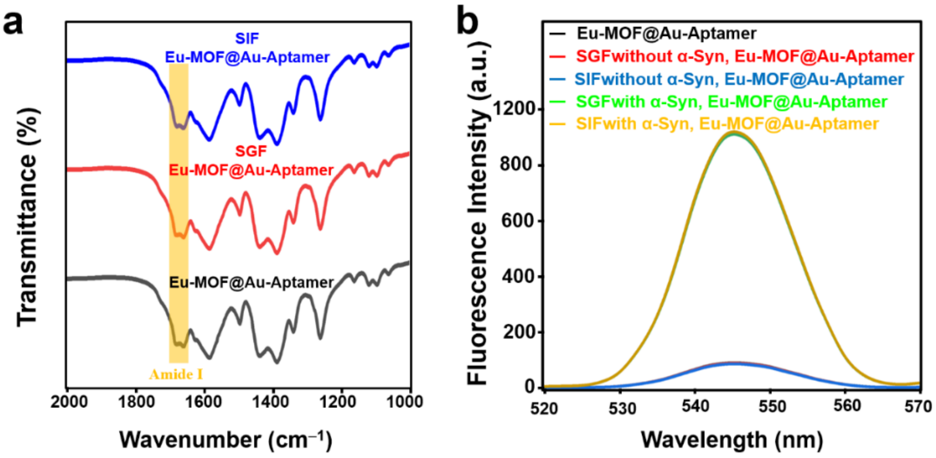

Figure S5. (a) FT-IR spectra of Eu-MOF@Au-Aptamer before and after exposure to simulated gastric fluid (SGF) and simulated intestinal fluid (SIF). (b) Fluorescence spectra generated by the "turn-on" fluorescence generation strategy before and after exposure to SGF and SIF.

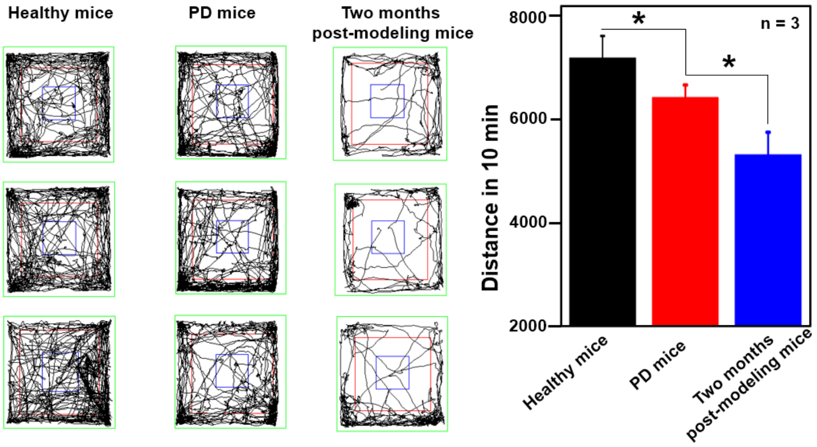

Figure S6. Travel distance observed during the open field test in mice from different groups.

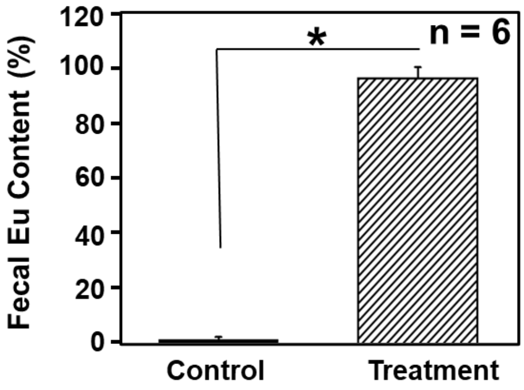

Figure S7. Eu contents of gut-microenvironment-triggered imaging sensor with untreatment control (Control) and treatment in fecal with PD mice. * denotes $P < 0.05$, which was considered statistically significant and $n = 6$ means that the experiment was repeated six times.

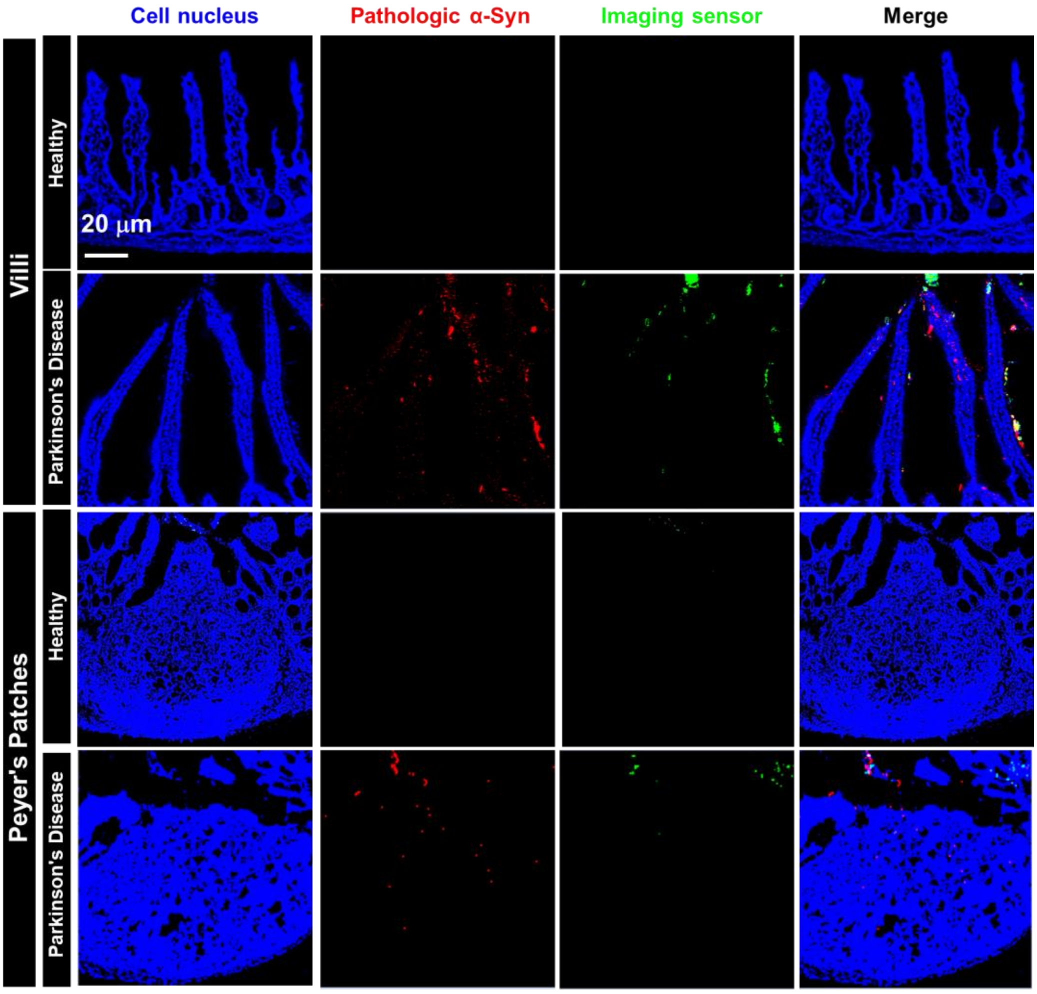

Figure S8. CLSM images of pathologic α-Syn expressed in the gut in test mice (healthy mice and PD mice) that had received gut-microenvironment-triggered imaging sensor treatments.

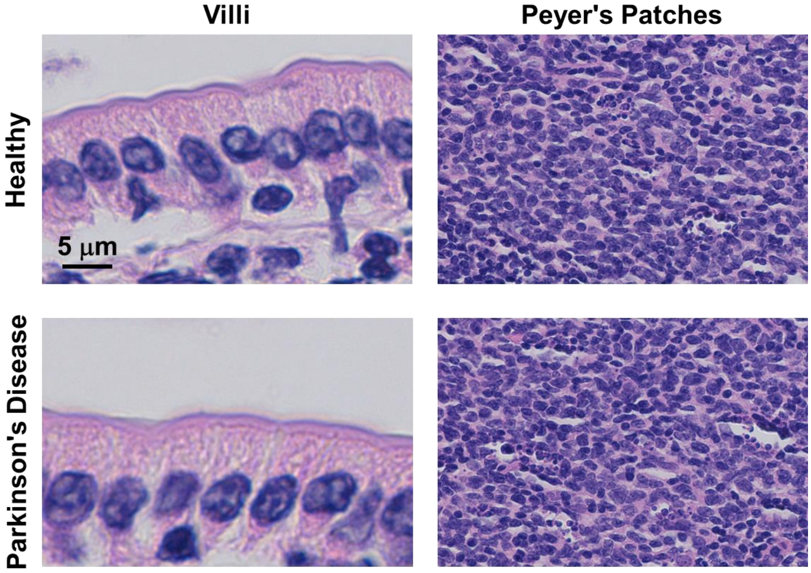

Figure S9. Histological photomicrographs of H&E-stained sections of gastrointestinal tract that were harvested from untreated mice (Control) and mice that had been treated with gut-microenvironment-triggered imaging agent (Treatment).

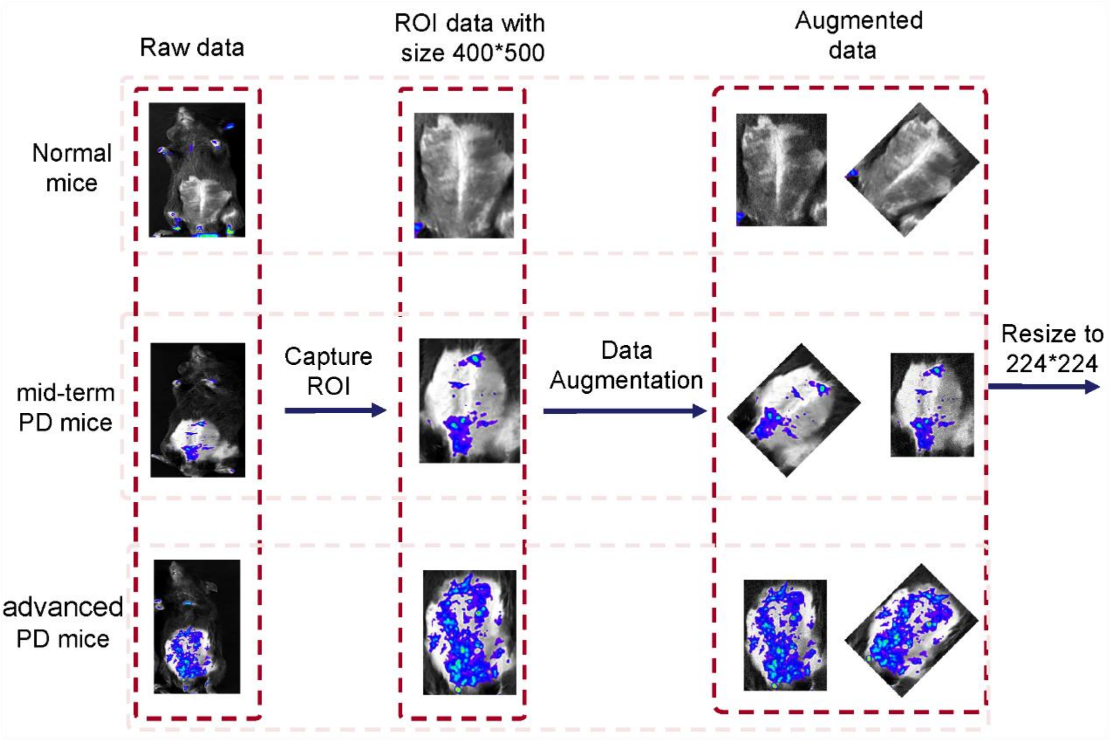

Figure S10. Framework of data preprocessing.

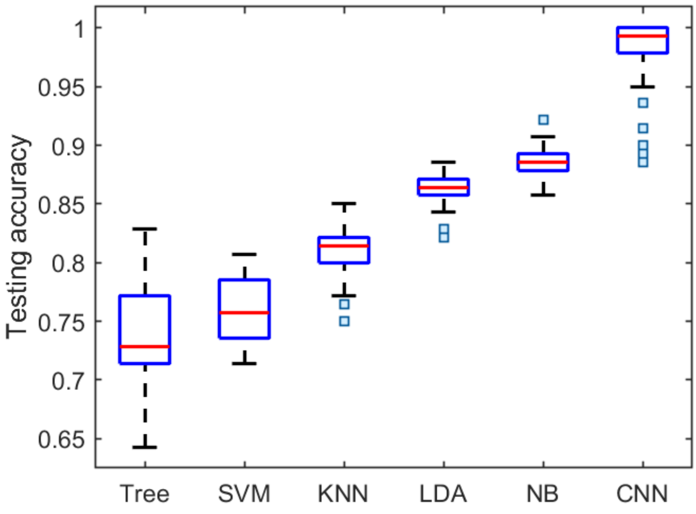

Figure S11. Testing accuracy comparison for the proposed algorithm (denoted by CNN) and the benchmark algorithms (Tree, SVM, KNN, LDA, NB).
